# Butyrate Shapes Immune Cell Fate and Function in Allergic Asthma

**DOI:** 10.3389/fimmu.2021.628453

**Published:** 2021-02-15

**Authors:** William Yip, Michael R. Hughes, Yicong Li, Alissa Cait, Martin Hirst, William W. Mohn, Kelly M. McNagny

**Affiliations:** ^1^School of Biomedical Engineering, The University of British Columbia, Vancouver, BC, Canada; ^2^The Biomedical Research Centre, The University of British Columbia, Vancouver, BC, Canada; ^3^Life Sciences Institute, The University of British Columbia, Vancouver, BC, Canada; ^4^Department of Microbiology and Immunology, The University of British Columbia, Vancouver, BC, Canada; ^5^Michael Smith Laboratories, The University of British Columbia, Vancouver, BC, Canada; ^6^Department of Medical Genetics, The University of British Columbia, Vancouver, BC, Canada

**Keywords:** butyrate, SCFA (short chain fatty acids), allergic asthma, epigenetics, microbiome, inflammation, cell fate and differentiation, HDAC inhibitor (histone deacetylase inhibitor)

## Abstract

The microbiome plays a fundamental role in how the immune system develops and how inflammatory responses are shaped and regulated. The “gut-lung axis” is a relatively new term that highlights a crucial biological crosstalk between the intestinal microbiome and lung. A growing body of literature suggests that dysbiosis, perturbation of the gut microbiome, is a driving force behind the development, and severity of allergic asthma. Animal models have given researchers new insights into how gut microbe-derived components and metabolites, such as short-chain fatty acids (SCFAs), influence the development of asthma. While the full understanding of how SCFAs influence allergic airway disease remains obscure, a recurring theme of epigenetic regulation of gene expression in several immune cell compartments is emerging. This review will address our current understanding of how SCFAs, and specifically butyrate, orchestrates cell behavior, and epigenetic changes and will provide a detailed overview of the effects of these modifications on immune cells in the context of allergic airway disease.

## Introduction

The gut microbiome is an intricate community composed of microorganisms from diverse groups of bacteria, fungi, protists, archaea, and viruses. Decades of research have revealed the significance of the gut microbiome in physiological processes, initially in regulating nutrition and metabolism (1) and, more recently, in the pathogenesis of respiratory, gastrointestinal, and neurological disease (2–7). These studies have revealed that perturbation of the microbiome can have acute and chronic effects on disease course and outcome. The intestinal microbiome collectively produces a wide variety of metabolites that may be involved in a spectrum of biological processes, ranging from immune defense and host immune cell interactions to inhibition of colonization by pathogenic bacteria (8). Short-chain fatty acids (SCFAs) are metabolites produced from the bacterial fermentation of indigestible fiber and amino acids in the intestinal lumen. In the human and murine gut, the three most abundant SCFAs are acetic acid (2 carbons), propionic acid (3 carbons), and butyric acid (4 carbons). The highest levels of SCFAs are found in the proximal colon, where they are either consumed locally as an energy source by intestinal epithelial cells (colonocytes) or transported across the gut epithelium and absorbed into the bloodstream (9, 10). Here we highlight the dynamic role of one SCFA, butyrate, in inflammatory immune cell responses in allergic airway disease.

## Source, Absorption, and Bioavailability of Butyrate

There are very few endogenously generated sources of butyrate and essentially all butyrate comes from the diet directly or via fermentation by commensal bacteria. Dairy products, especially butter, contain butyrate but these sources of the metabolite are paltry compared to the butyrate produced by intestinal commensal bacteria from non-digestible dietary fiber (11). In humans, this fermentation primarily takes place in the proximal large intestine by butyrate-producing Firmicutes phylum, including *Ruminococcaceae, Lachnospiraceaes, Erysipelotrichaeceae* and *Clostridiaceae* [reviewed in (11)].

**Figure 1:**
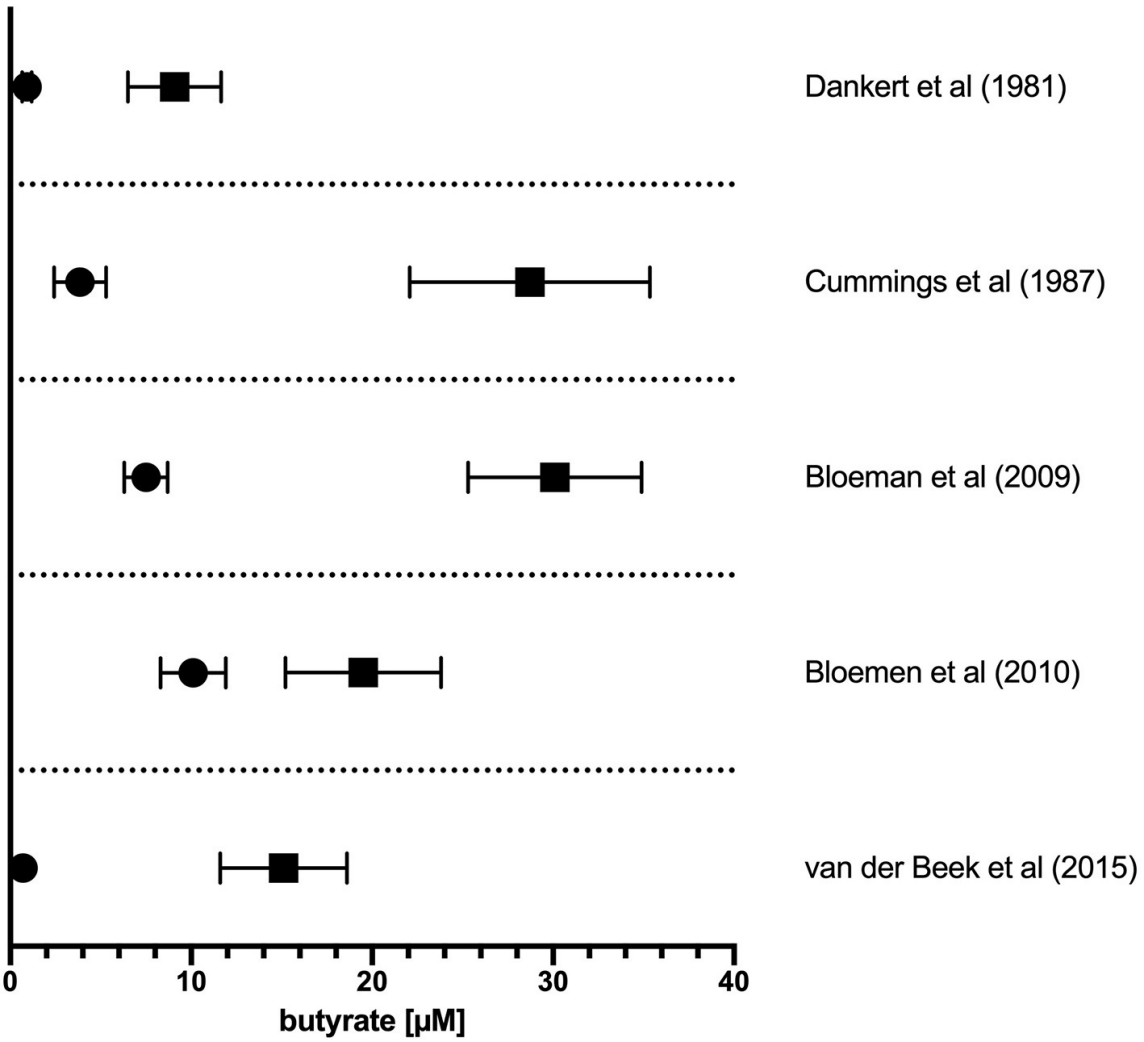
Concentration of butyrate in portal vein and peripheral circulation in humans. The reported concentration of butyrate in plasma sampled from the portal vein plasma (solid squares) or peripheral circulation (solid circles). Values from original references were plotted and are displayed as means ± SEM as reported in original articles.

**Figure 2:**
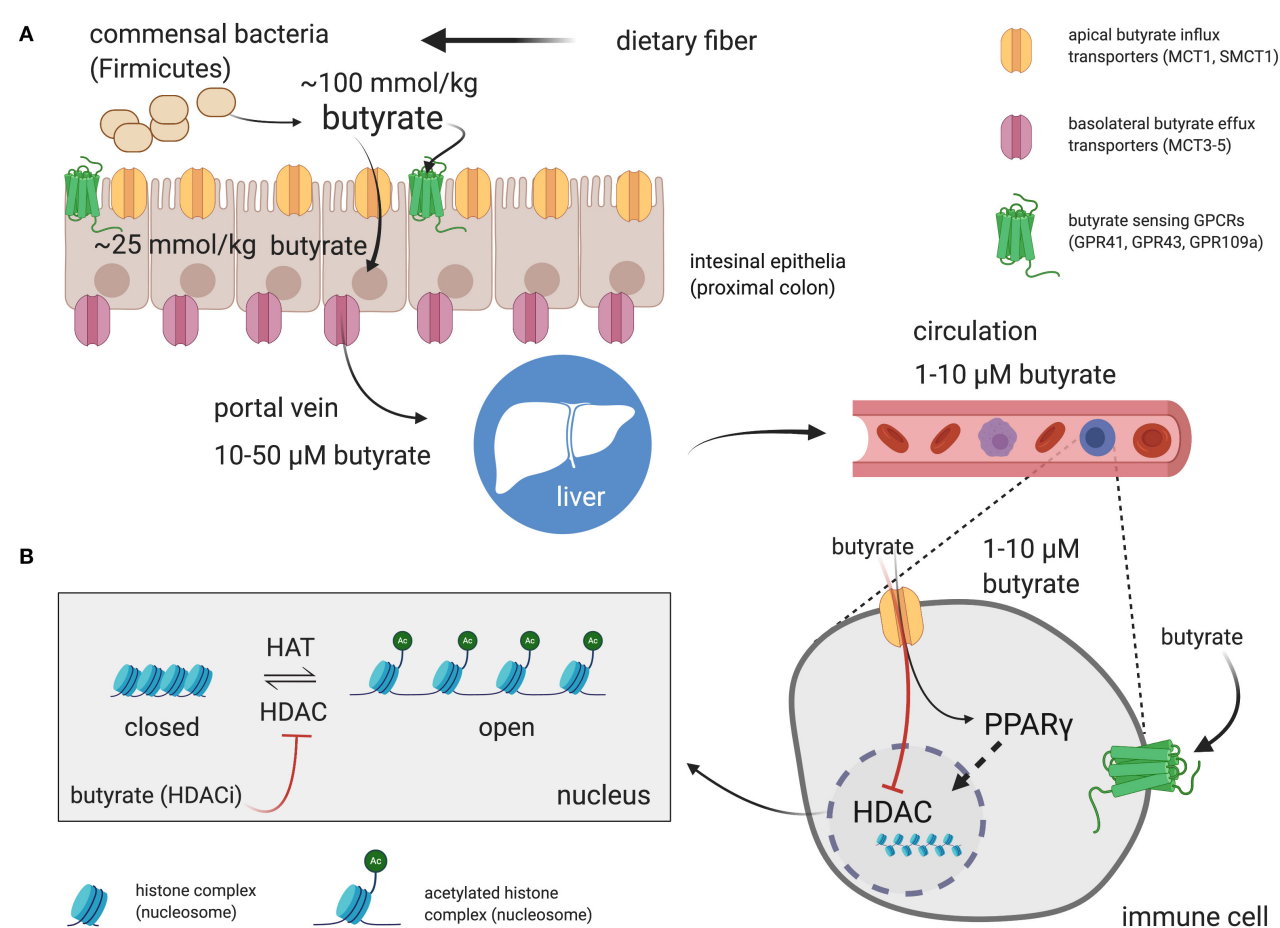
Butyrate production in the intestinal lumen, absorption in the gut, and peripheral distribution. **(A)** Butyrate is produced from the fermentation of dietary fiber by commensal bacteria (mainly Firmicutes phylum) in the gut lumen. Butyrate can stimulate butyrate-sensitive G protein coupled receptors (GPCRs) expressed on luminal epithelia. However, most of the butyrate is efficiently absorbed by colonocytes through H^+^- or Na^+^-coupled monocarboxylate transporters (MCT1 and SMCT1, respectively) expressed on the apical surface of intestinal epithelia. Most of the butyrate is consumed by colonocytes for energy. The remainder is passed through the basolateral membrane into the liver portal system via monocarboxylate transports (MCT3-5). Butyrate then transits to the liver and is absorbed by hepatocytes. Any remaining butyrate not used as an energy source by hepatocytes is then distributed through the circulation to peripheral tissues. The effects of butyrate on immune cells, and most other cell types are mediated through direct activation of surface GPCRs or, following influx into the cell by activation of peroxisome proliferator-activated receptor gamma (PPARγ) or inhibition of butyrate-sensitive histone deacetylase (HDAC) isoforms. **(B)** Most of the reported effects of butyrate on immune cells discussed in the review are dependent on HDAC inhibitory activity of butyrate. Side chain lysine in histone complexes (nucleosomes) in condensed, closed, chromatin are acetylated by histone acetyltransferases (HATs) to provide access for transcription machinery. HDACs remove acetyl groups from histone lysines to promote chromatin condensation and, in general, attenuate gene transcription at targeted loci. Butyrate is the most potent “endogenous” HDAC inhibitor (HDACi) and thereby promotes open chromatin and encourages active transcription. The shown concentrations are those reported for butyrate in the indicated compartments in humans. Created with https://biorender.com/.

The literature-reported concentrations of SCFAs (including butyrate) in blood circulation and tissues varies, likely owing to differences in diet and disease state as well as methods of tissue/fluid collection, processing, and assay for these volatile SCFA. Nevertheless, the measured order of magnitude of butyrate concentrations in tissues are consistent (Figure 1). The physiological concentration of butyrate in humans is highest in the large intestinal lumen (~100 mmol/kg chyme) (12) and intestinal tissue (~25 mmol/kg tissue in cecum, ascending, and transverse colon) (13). Microbially-produced butyrate in the mammalian gut lumen is transported across the apical mucosal surface of colonocytes via the proton-coupled monocarboxylate transporter isoform 1 (MCT1, gene name *SLC16A1*) or the Na^+^-coupled monocarboxylate transporter 1 (SMCT1, gene name *SLC5A8*) (9, 14). The efflux of butyrate into blood circulation is accomplished by monocarboxylate transporters (MCT3-5) located at the basolateral surface of colonocytes (14) However, as butyrate is an important source of fuel for colonocytes, much of the absorbed butyrate is metabolized for energy within colonocytes (14). As such, the systemic butyrate concentration rapidly declines with increasing distance from the liver portal system (10–50 μM in portal vein plasma) so that, in circulation and peripheral tissues, butyrate concentrations in human blood circulations are ~1–10 μM (13, 15–19). Butyrate concentration in mouse circulation is typically reported to be up to 100 μM (18, 20, 21). Non-metabolized butyrate is then transported to the liver through the blood where it is absorbed again via hepatocyte MCT1 and SMCT1 and largely metabolized. Absent a concentrating mechanism, butyrate levels in organs other than the colon and liver are likely in the low μM range as well. MCT1 and SMCT1 are widely expressed transporters and therefore also allow uptake of butyrate directly into most cell lineages including immune cells (9). Although passive diffusion of non-ionic butyrate (protonated form) has been proposed as an alternative method for butyrate uptake, it is unlikely to contribute significantly to intracellular butyrate concentrations (22) (Figure 2).

In large part because of the availability of butyrate, most evidence for its effects on inflammatory cell function, and the study of the underlying molecular mechanisms, have focused on tissues that are exposed to mM-range concentrations of butyrate *in vivo* (i.e., intestinal epithelia) or purified cells exposed to these concentrations *in vitro*. In normobiotic humans and mice, direct oral dietary supplementation of butyrate will only marginally increase circulating or, presumably, peripheral tissue butyrate concentration (18, 20, 21, 23). A single large dose (maximum toleration) of butyrate will transiently spike circulating butyrate levels in mice to ~10 mM, but levels return to baseline (~100 μM) in 30 min (intravenous administration) or ~3 h (enteral administration) (24). Nevertheless, evidence for the effect of butyrate on inflammatory disease pathophysiology outside of the liver and intestine is mounting.

## Butyrate Sensing Mechanisms

### G Protein-Coupled Receptors

Butyrate is sensed by cells via three known cell-surface receptors of the G protein-coupled receptor (GPCR) class: GPR41/FFAR3, GPR43/FFAR2, and GPR109A/HCAR2 [reviewed in (9)]. These receptors vary in their sensitivity and response to butyrate, they bind other ligands (including other SCFAs), and they have both overlapping and distinct tissue expression patterns (9). GPR41 and GPR43 are expressed on immune cell lineages and are also activated in response to acetate, propionate, butyrate, and other SCFAs. Conversely, GPR109A, which is also expressed on immune cells, responds strongly to both butyrate and nicotinate but not acetate and propionate. Critically important for the consideration of butyrate's role in immune cell function, the half maximal effective concentration (EC_50_) of butyrate for GPR41, GPR43, and GPR109A is in the high μM to low millimolar (mM) range [reviewed in (25)]. Thus, one would predict the circulating concentration of butyrate in most peripheral tissues other than liver and intestinal lumen may be too low to ***potently***activate these receptors.

### PPARγ Agonist

Peroxisome proliferator-activated receptors (PPARs) are a family of ligand-activated transcription factors that are activated by fatty acids and eicosanoids (26). PPARγ1 is expressed highly in adipose tissue, the large intestine and immune cells (27, 28). PPARγ can be activated by butyrate but not acetate and propionate (29). Activation of PPARγ has broad anti-inflammatory effects in many cell types. In intestinal epithelial cells, at concentrations of 0.01–1 mM, butyrate induces activation of PPARγ, and promotes epithelial barrier integrity (30). Thus, although immune cells do express PPARγ, effective activation of PPARγ may be restricted to the intestinal epithelium and liver where butyrate concentrations provide adequate exposure.

### HDAC Inhibitors

Butyrate is a non-selective and potent endogenous inhibitor of “classical,” Zn^2+^-dependent class I, II, and IV histone deacetylases (HDACs) [reviewed in refs (31–33)]. HDACs are enzymes that hydrolyze *N*-acetyl groups from lysine residues of protein substates, in particular, acetylated side-chain lysines in the histones of chromatin nucleosome complexes. In general, acetylation of histones by histone acetyltransferases (HATs) enhances chromatin accessibility and facilitates transcription. HDACs reverse the process by driving a return to a silent state typical of more condensed chromatin. The specific regulation of gene activation is much more complicated of course, and HAT and HDACs are only part of the control mechanisms. Overall, inhibition of HDACs by butyrate is expected to promote gene transcription from targeted chromatin. HDACs also have non-histone targets including the transcription factors forkhead box P3 (FoxP3) and several others (33–36). Acetylation of these transcription factors has been reported to regulate their stability, alter protein-protein interactions, affect subcellular localization, and modify transcriptional activating functions. Thus, HDAC modification of these non-histone targets would also be expected to alter cell activity independently (or in conjunction) with regulation of gene expression (32, 33). It is not always clear which HDAC isoforms are responsible for direct deacetylation of these targets. Finally, many HDAC isoforms participate in multi-protein regulatory complexes and thus may have activity in regulation of cell processes independent from their deacetylase catalytic domain (33). HDAC inhibitors (HDACi) have a varied effect on cell cycle, differentiation, and cell death (apoptosis, necrosis and autophagy) (33). The class I isoforms (HDAC1, 2, 3, & 8) are ubiquitously expressed whereas class II isoforms (HDAC4, 5, 6, 7, 9, & 10) and the sole class IV isoform (HDAC11) have more restricted tissue distribution including expression in some immune cell subsets (32, 33). Although the activity of class I isoforms is largely (but not completely) restricted to the nucleus, class II and IV isoforms shuttle between the nucleus and cytoplasm. Butyrate inhibits the activity of the class I HDACS (1, 2, 3, & 8) and class IIa HDACs (4, 5, 7, & 9). HDAC11 expression itself is also potently induced in human myeloid cells in response to butyrate exposure (37). The half-maximal inhibitory concentration (IC_50_) of butyrate for HDACs *in vivo* is reported to be <1 mM depending on the type of assay and substrate (33, 38). The potency of butyrate is likely different for each of the responsive HDAC isoforms. Indeed, in a cell-free enzymatic assay using recombinant HDACs (1–4, 6–8, & 10), butyrate most potently inhibited class I HDACs 1, 2, 3, & 8 with IC_50_ values ~10–20 μM (39). Notably, although butyrate is a potent natural inhibitor of HDAC enzymatic activity, it is 10^3^-10^6^-fold less potent than known pharmacological inhibitors including Entinostat and Panobinostat (27, 40).

## Effects of SCFA on Allergic Lung Disease and Asthma

Analysis of SCFA fecal concentrations of 1-year-old infants in the European PASTURE study shows that children with the highest butyrate and propionate concentrations (≥95-percentile) were about half as likely to be sensitive to allergens at age 6 (41). Higher acetate concentrations were not correlated with lower incidence of atopy in this study. However, in another study, higher serum acetate in pregnant women during late phase pregnancy was associated with fewer doctor visits for cough/wheeze or parent-reported wheeze in the first year after delivery (42).

In animal model studies, mice fed SCFA (butyrate, propionate, or acetate) exhibit less severe disease in a model of allergic airway disease than those raised on a normal diet (43). Exogenous butyrate administered orally to adult BALB/c mice prior to disease induction attenuates severity measures of ovalbumin (OVA)-induced asthma including airway hyperresponsiveness (AHR), infiltration of eosinophils into the bronchoalveolar fluid and the frequency of CD25+FoxP3+ T regulatory cells (Tregs) in the lung tissue (41). Importantly, oral administration of these same SCFA to pregnant and nursing BALB/c dams also attenuated some symptoms of allergic inflammation in weaned adult offspring including eosinophil BAL-infiltrates and an elevated frequency of CD25+FoxP3+ Tregs. This treatment approach failed to attenuate AHR, however (41). Similarly, C57Bl/6 mice fed a high-fiber diet or regular diet supplemented with acetate displayed attenuated disease severity in a house-dust mite (HDM) model of allergic airway disease (AAD) (42). This included attenuated AHR, BAL, and lung immune cell infiltrate (including eosinophils), goblet cell hyperplasia, and serum IgE concentration. Many of these same benefits were observed in adult C57Bl/6 that had been delivered by Cesarean section and cross fostered with mothers on a regular diet from dams that had received high fiber diet or acetate supplementation during pregnancy (E13-E18) (42). In these experiments, the high fiber diet significantly increased acetate concentrations in the feces and serum but did not alter fecal or serum butyrate concentration (42). Conversely, treatment of mice with vancomycin, an antibiotic that depletes butyrate-producing intestinal bacteria, exacerbates AAD (44). Oral supplementation of vancomycin-treated mice with SCFA (mixture of butyrate, acetate, propionate) reverses this effect and ameliorates disease severity (43). We have found that both, the vancomycin-induced severe AAD, and the restorative effects of SCFA-supplementation require early life application to alter the subsequent adult allergic responses. Similarly, there is likely a limited window of opportunity to alter later-life allergic responses in humans associated with early-life (or prenatal) exposure to microbial metabolites including SCFAs (44). These long-lasting effects would, accordingly, suggest SCFAs function by altering, long-term, the trajectory, development, and function of blood cell precursors in addition to any potential effects on terminally differentiated mature cells.

## Role of Butyrate In Human Allergic Asthma

**Figure 3:**
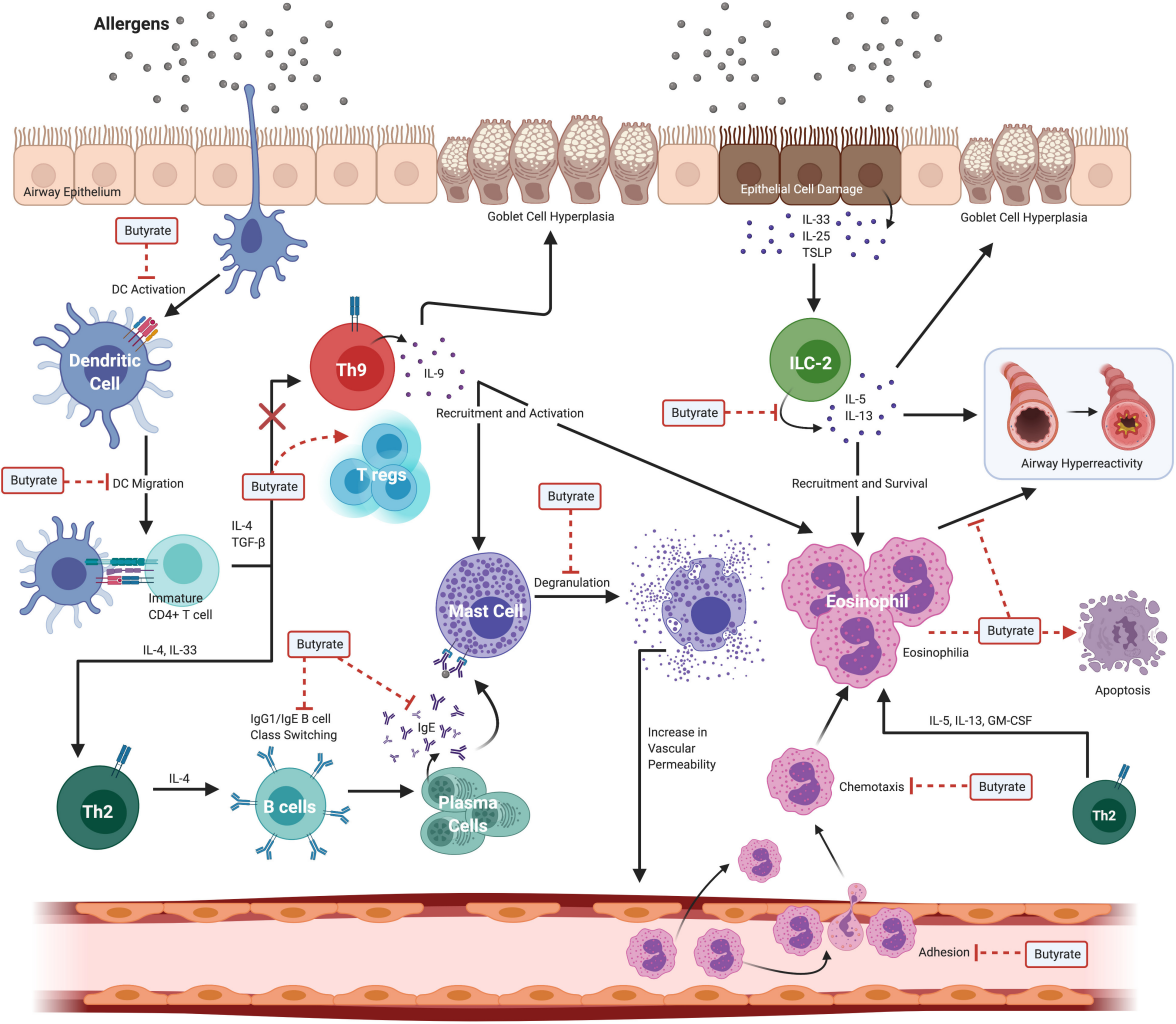
Widespread effects of butyrate on immune cells in allergic asthma. Allergic asthma is a complex inflammatory disease with several immune cells involved in the pathogenesis. Exposure to an allergen induces eosinophilia, airway hyperreactivity, and goblet cell hyperplasia. These effects are collectively driven by dendritic cells (DCs), Th2 cells, Th9 cells, ILC2s, B cells, mast cells, and eosinophils. Butyrate ameliorates allergic asthma by modulating various steps in pathways of different immune cell compartments. Butyrate suppresses both DC activation and migration to local lymph nodes where activated DCs function to stimulate immature/naive CD4+ T cells to polarize to the Th2 lineage. In the B cell compartment, butyrate suppresses both B cell isotype class switching and plasma cell differentiation leading to decreased levels of circulating IgE. Subsequent binding of allergens and cross-linking of surface bound IgE to Fc receptors expressed on mast cells induces degranulation; however, butyrate inhibits IgE-mediated mast cell degranulation. In the Th9 cell lineage, butyrate functions to divert the fate of naïve CD4+ T cells from Th9 to FoxP3+ regulatory-T cells (T regs) effectively promoting a regulatory phenotype. In ILC2s, butyrate suppresses the secretion of IL-5 and IL-13 cytokines that have downstream effects on eosinophils. Butyrate inhibits both the adhesion of eosinophils to the blood vessel endothelium, chemotaxis in response to CCL24, and directly promotes eosinophil apoptosis. Created with https://biorender.com/.

The severity of allergic asthma in mouse models and humans appears to correlate with the presence of butyrate producing intestinal commensals and, in some instances the presence of butyrate directly. Despite this association, delineating the mechanisms and testing the causal role for butyrate in the attenuation of atopy and asthma has proven difficult. Part of the difficulty is that butyrate has wide-spread functions in intestinal homeostasis that may have an indirect impact on peripheral immune functions (45). In addition, butyrate has the potential to epigenetically alter cell fate(s) so that the biological effects of butyrate exposure may be observed long after the initial exposure (33, 45, 46). Thus, when considering its effects, one may need to consider that alterations in cell function may reflect an exposure to butyrate that occurred much earlier in the life history of a cell or lineage. Additionally, the presence of butyrate and the commensals that produce it *in vivo*, may also herald exposure to several other potentially immune modulating metabolites produced by the same bacteria that may contribute to the ultimate phenotype of the individual or experimental animal (6, 47). Finally, studies that measure fecal SCFA and butyrate often fail to report blood plasma concentrations, which are difficult to accurately measure. A recent study showed that in adult normobiotic human males receiving twice daily 2 g sodium butyrate for 4 weeks, the plasma levels of butyrate were not significantly altered compared to pre-treatment levels (23). These treatments did, however, have a marginal anti-inflammatory effect on peripheral blood mononuclear cells (PBMCs) stimulated with innate ligands (23). Figure 3 illustrates various effects of butyrate on immune cells in allergic asthma.

Despite evidence of oral SCFA administration attenuating allergic inflammation in murine studies (41, 43) treatments to successfully prevent the development of allergies in human remain unclear. However, the PASTURE study suggests that infants on a diet composed of yogurt, fish, vegetables, and/or fruits have an increased level of fecal butyrate. Children with the highest level of butyrate were less likely to develop asthma and food allergy (41). Cait et al. (48) found that bacterial butyrate production protects children from developing atopy. Specifically, the CHILD study showed that the genes encoding for butyrate fermentation and carbohydrate-active enzymes (CAZymes) which degrade human milk oligosaccharides (HMOs) were depleted in children at age 3 months. Therefore, butyrate production genes may be used as biomarkers to monitor infants who have the genetic propensity (familial history) or other risk factors for atopy.

## Effects of Butyrate on Immune Cell Subsets in Allergic Asthma

### Eosinophils

Eosinophil influx into the lung parenchyma is a hallmark feature of the most common form of allergic asthma. During allergic inflammation, interleukin 5 (IL-5), IL-13, and granulocyte macrophage colony-stimulating factor (GM-CSF) secreted by Th2 cells and innate lymphoid cells (ILCs) type 2 promote the survival of eosinophils (49–51). Recent *in vitro* studies using human peripheral blood eosinophils show that butyrate promotes eosinophil apoptosis and limits their adhesion to endothelial cells under flow in response to CCL24 (eotaxin-2). Butyrate, but not propionate, also inhibited eosinophil migration in response to CCL24 (52).

Both GPR41 and GPR43 transcripts are expressed by human eosinophils (52) but only GPR43 appears to be translated and expressed on the cell surface (53, 54). Unlike acetate and propionate, butyrate does not induce Ca^2+^ release or stimulate reactive oxygen species (ROS) production via GPR43 (52). However, butyrate (3–10 mM) and propionate (10 mM) induce apoptosis of human eosinophils from allergic-donors 18 h after exposure (52). Surprisingly, this effect appears to be independent of GPR41/43 receptor stimulation and instead depends on influx of these SCFA into eosinophils via monocarboxylate transporters (52). Exposure of eosinophils to butyrate or propionate induces the intrinsic apoptotic pathway including morphological changes in cell size and nucleus structure, mitochondrial depolarization, caspase-3/7 activation, and reduced expression of pro-survival factors myeloid cell leukemia 1 (MCL-1) and B cell lymphoma extra-large (BCL-XL) (52). Importantly, only eosinophils isolated from allergic donor peripheral blood exhibit sensitivity to butyrate- or propionate-induced caspase-3/7 activation and apoptosis (52). Intriguingly, eosinophils from non-allergic donors require priming with IL-5 before butyrate or propionate promotes caspase-3/7 activation (52). This suggests that these SCFA exert their effects through inhibition of pro-survival pathways regulated by IL-5 in activated eosinophils.

Butyrate and propionate regulate eosinophil survival and migration via inhibition of class IIa HDACs. Indeed, both a pan-specific HDACi (trichostatin A (TSA)) and a class IIa-specific HDACi (MC1568) also promote dose-dependent apoptosis of eosinophils (52). Both propionate and butyrate induce acetylation of histone 3 (H3) starting at 3 h after exposure and peaking at 18 h (52). Notably, the biological effect of these SCFAs on eosinophils is most robust selectively after 18-h, coinciding with epigenetic regulation of gene expression by HDAC (52). Exposure of allergic donor eosinophils to butyrate is associated with decreased transcript and protein expression of homing, extracellular matrix, and chemotactic receptors including integrin alpha-4 (CD49d), CD44, and CCR3, respectively (52). Notably, butyrate and propionate also potently reduce IL-5 receptor alpha (*IL5RA*) transcript expression (52).

The *in vitro* assays outlined above required low mM concentrations of butyrate to affect cell activity. Systemic administration (intravenous) of sodium butyrate to mice at a dose of 1 g/kg achieves transient (30–60 min) plasma butyrate concentrations in the 1–10 mM range (24). In an OVA-induced mouse model of asthma, daily administration of 1 g/kg systemic butyrate (i.p.) during the challenge phase resulted in decreased eosinophilia and lower concentrations of type 2 cytokines (IL-4,−5,−13) in the bronchoalveolar lavage fluid (BALF) and attenuated airway hyperresponsiveness (52). Thus, mM plasma concentrations of butyrate, even transiently, can alter allergic eosinophilia and type 2 allergic response *in vivo*. It is important to note that these experimental butyrate concentrations are not normally achievable through microbially-produced butyrate.

### Mast Cells

Mast cells are key effector cells in allergic inflammation and can initiate and propagate inflammation in atopic disease. Circulating immunoglobulin isotype E (IgE) binds to high-affinity Fc receptors expressed on the surface of mast cells. The subsequent binding of allergens and cross-linking of surface-bound IgE triggers the release of preformed granules containing proteases, lysozymes, an array of cytokines, histamine, and eicosanoids. The release of histamine, a vasoactive amine, increases vascular permeability that leads to inflammation. Mast cells are found near both vascular and lymphatic vessels and are in mucosal and barrier tissue sites such as the gastrointestinal tract, lung airways, and skin where they play a pivotal role in driving allergic inflammation [reviewed in (55, 56)].

Butyrate treatment (5 mM, 24 h exposure) of precision cut lung slices harvested from OVA-sensitized guinea pig demonstrated greatly decreased allergen-induced histamine release and attenuated airway contraction (57). In addition, butyrate and propionate (but not acetate) potently inhibit IgE- and non-IgE mediated human and mouse mast cell degranulation and IL-6 secretion. These effects are independent of GPR41, GPR43, and PPARγ but depend on butyrate's function as an HDACi. Butyrate exposure reduces the expression of transcripts for Bruton's tyrosine kinase (*BTK)*, spleen tyrosine kinase (*SYK)*, and linker of activated T cells (*LAT*) in human mast cells. These are tyrosine kinases that are well-known to propagate IgE receptor signaling pathways (57) upstream of degranulation and cytokine secretion. In mice, butyrate exposure reduces the expression of levels of BTK and SYK but not LAT proteins. ChIP-Seq analysis of histone 3 lysine 27 (H3K27) acetylation in butyrate-treated primary human mast cell cultures revealed increased global H3K27 acetylation levels as would be expected in the presence of a pan-specific HDACi (57). Curiously, H3K27 acetylation levels near the transcriptional start sites of *BTK, SYK*, and *LAT* are markedly ***reduced***after butyrate treatment (57). This suggests that, although butyrate acts on mast cells by evoking broad histone acetylation (as an HDACi), it also selectively promotes deacetylation near promoter regions of genes associated with FcεRI-mediated mast cell activation with the consequence of attenuated gene expression (57). The mechanism behind how butyrate evokes selective deacetylation remains unexplored.

### Regulatory T Cells

Butyrate (100–125 μM) and, less potently, propionate (> 1 mM) promote transforming growth factor beta (TGF- β-dependent FoxP3+ regulatory T cell polarization. In this scenario, naïve CD4+ T cells mature into Tregs in response to TCR activation in the presence of Flt3 ligand (Flt3L)-elicited dendritic cells (DCs) (58). This enhanced Treg polarization is partly explained by butyrate-induced enhanced acetylation, in CD4+ T cells, of the *FoxP3* promoter and conserved non-coding sequence 1 (CNS1), an intronic enhancer essential for extrathymic Treg differentiation (58). In addition to its direct effects on T cells, butyrate also enhanced chromatin acetylation (specifically histone 3 (H3)) in the co-stimulatory Flt3L-DCs that provide exogenous help to drive T cell polarization. Furthermore, expression of *Relb*, a transcription factor known to promote DC activation is suppressed by butyrate (58) and this has previously been shown to promote Treg polarization (59, 60). Several non-histone proteins have been identified as direct targets of HDAC deacetylase activity, many of which are transcription factors (36, 61). Hyperacetylation of FoxP3 prevents ubiquitin-dependent degradation and thus stabilizes this protein and, in addition, enhances its DNA binding or association with other HDACs and TFs (34, 35, 61–63). Arpaia et al. (58), showed enhanced levels of acetylated FoxP3 protein in the presence of butyrate. Importantly, direct acetylation of FoxP3 by butyrate-sensitive HDACs has been demonstrated, and HDAC9, a known effector of FoxP3 acetylation is ***not***butyrate-sensitive. Thus, butyrate is most likely supporting FoxP3 expression rather than directly altering its acetylation state.

In aggregate, the net effect of butyrate on naïve T cells and co-stimulatory DCs is enhanced generation of extrathymic Tregs and a dampened pro-inflammatory milieu. Notably, expression of GPR109A in DCs was not involved in their Treg polarizing activity in response to butyrate (58). In mice rendered dysbiotic by an antibiotic cocktail consisting of ampicillin, vancomycin, neomycin and metronidazole (AVNM), and thus lacking SCFA-fermenting commensal bacteria, FoxP3+ Treg levels could be restored in the spleen and intestinal lymph nodes by supplementing with SCFA in drinking water (36 mM of each) (58). This SCFA supplement returned serum butyrate concentrations of AVNM dysbiotic-mice to levels comparable to normobiotic mice (~5–6 μM) (58). It is notable that Treg levels in both the intestinal lymph nodes and the spleen were restored with SCFA supplementation, suggesting that relatively low concentration of butyrate in the blood may be sufficient to be immunomodulatory in peripheral tissues and not just the intestinal lumen (58).

### CD4^+^ T_H_2 Cells/Dendritic Cells

Dendritic cells (DCs) are key players in the initial recognition of antigen and serve to bridge the innate and adaptive arms of the immune system in allergic inflammation. DCs are professional antigen presenting cells and are responsible for the uptake of the antigen at barrier sites and the subsequent migration to the local lymph nodes where they present antigen and provide the necessary signals required for T cell activation in the adaptive immune response. For T cells to be activated, DCs must present processed antigen in the form of peptides on MHC class II molecules and provide the costimulatory molecules CD80 and CD86.

Butyrate (2 mM) suppresses lipopolysaccharide (LPS)-induced activation of human monocyte-derived DCs (moDCs) *in vitro* by limiting upregulation of co-stimulatory markers CD40, CD80, and CD83 and reducing DC metabolic activity (64). In addition, exposure of moDCs to butyrate alters the ability of moDCs to promote the polarization of naïve CD4+ T cells toward the IL10-producing type 1 regulatory T cell lineage (FoxP3^lo^ Tr1) (64). Butyrate induces expression of retinaldehyde dehydrogenase (*ALDH1A1*) in moDCs with a consequent increase in retinoic acid (RA) production. The resultant RA functions in an autocrine and paracrine fashion on naïve CD4+ T cells to promote FoxP3^lo^ Tr1 polarization and IL-10 production (64). IL-10 from these cells leads to a subsequent suppression of effector T cell proliferation in the inflamed tissue (64). Interestingly, this butyrate induced effect on moDCs-dependent Tr1 polarization requires both HDACi and GPR109A signaling activity in moDCs (64). Only the combination of niacin (a natural ligand of GPR109A) and the HDACi TSA can promote moDCs Tr1-priming at levels comparable to butyrate alone (64).

More recently, independent studies have shown that butyrate can have a more selective role on DC function in models of allergic diseases. Previously, we reported that the exposure of DCs to butyrate attenuates both DC activation and chemotaxis (43). Transcriptomic analysis of isolated splenic DCs incubated with butyrate (40 mM) and activated by LPS *ex vivo* reveals altered gene expression profiles in two key pathways involved in allergic disease susceptibility: activation of lymphocytes and DC trafficking (43). As stated above, butyrate has been shown to attenuate DC activation by reducing the expression of costimulatory molecules CD80 and CD86. *Ex vivo* studies using isolated DCs from naïve mice shows that butyrate also reduces the expression of CD80 and CD86 after LPS stimulation while, DCs isolated from vancomycin-treated mice in the absence of butyrate show increased expression levels (43). Thus, butyrate-induced reduction in the costimulatory molecules on DCs limits the ability of DCs to activate T cells generally and, more specifically, dampens their ability to polarize T cells to the Th2 lineage.

Butyrate also reduces the chemotactic potential of DCs by attenuating the responsiveness of DCs to CCL19 chemokine (43). *In vitro* studies using a transwell chemotaxis assay show that DCs isolated from naïve mice and incubated with butyrate exhibit decreased chemotaxis in response to CCL19 as compared to DCs incubated without butyrate (43). DCs isolated from vancomycin-treated mice however exhibit increased migration. In a papain-induced mouse model of allergic asthma, DQ-OVA was used to track the migration of DCs *in vivo* from the airways upon the intranasal administration of papain (43). Results from this experiment indicate that vancomycin-treated mice challenged with papain had significantly higher numbers of DCs in the mediastinal lymph nodes compared with naïve challenged mice. Furthermore, the administration of a mixture of SCFAs (butyrate, acetate, and propionate) reduces the trafficking of DCs in vancomycin-treated mice to levels comparable to control mice. In aggregate these studies would argue that butyrate has a profound effect on the adaptive immune response to allergens by dampening the ability of DCs to migrate to the draining lymph nodes and subsequently prime Th2 polarization.

### CD4^+^ T_H_9 Cells

T helper 9 (Th9) cells are a subset of IL-9 producing CD4+ T cells developmentally related to Treg and Th2 subsets (65). Th9 cell development from naïve CD4+ T cells is highly dependent on the presence of both IL-4 and TGF-β (65, 66). In general, Th9 cells facilitate immune processes involved in parasitic clearance and anti-tumor response (65–67). More recently, the role of Th9 cells in the immunopathology of allergic lung diseases and autoimmunity has been investigated in animal models (68). Th9 cells promote allergic lung inflammation by orchestrating the recruitment and activation of eosinophils and mast cells (in direct response to IL-9 secretion) and by stimulating mucus production by lung epithelial cells (69, 70).

In *in vitro* polarization assays, butyrate (0.25 mM) and less potently, propionate (0.25 mM), divert the fate of naive CD4+ T cells from Th9 cells to FoxP3+ Tregs under conditions that favor Th9 polarization and reinforce FoxP3 expression under Treg polarizing conditions (68). In other words, butyrate negatively modulates Th9 cell differentiation by inducing robust FoxP3 expression during naïve CD4+ T cell polarization. FoxP3 expression directly suppresses IL-9 production in CD4+ T cells (68). As such, the anti-Th9 effect of butyrate is in part due to an effect on cell fate rather than a direct regulatory interaction between butyrate and Th9 cells. In fact, the dampening effect of butyrate on IL-9 production in naïve CD4+ T cells is not observed in mature Th9 cells, which would be expected to promote allergic inflammation (68).

In an OVA-model of allergic lung inflammation, administration of butyrate (250 μL of 1 M butyrate, i.p.) to C57Bl/6 mice during both the systemic OVA sensitization and challenge phase attenuated disease severity by limiting eosinophilia (68). Systemic butyrate treatment weakly, but significantly, reduced the frequency (2.8 2%) of IL-9 producing CD4+ T cells in the lung. Adoptive transfer of Th9 cells, but not naïve CD4+ cells, or administration of IL-9 reversed the disease-dampening effect of butyrate (68). Together, these results suggest that the presence of butyrate *in vivo*, skews Th polarization away from pro-inflammatory T cells fates in favor of suppressing fates (Tregs) and thus ameliorates allergic lung disease. Notably, the butyrate dose used to observe these phenotypes (equivalent to about 0.8–1 g/kg) likely produces a transient (up to 60 min) spike in plasma butyrate to the 1–10 mM range.

### Type 2 Innate Lymphoid Cells

ILCs are a distinct population of hematopoietic cells that arise from common lymphoid progenitors (CLP). ILCs are part of the innate immune system and they function to orchestrate immunity, inflammation, and tissue repair (71–73). Their role in mucosal immunity has been studied extensively. Subsets of ILCs (natural killer (NK) cells, ILC1s, ILC2s, and ILC3s) are defined by differential expression of cell surface proteins, transcription factors and effector cytokines and closely mimic T cells in form and function (cytotoxic T cells, Th1, Th2, and Th17 cells, respectively). In fact, the most prominent distinguishing characteristic between T cells and ILCs is the distinct lack of a TCR in the latter. Thus, they can, for all intents and purposes, be considered innate counterparts of these highly specialized adaptive immune subsets (74, 75). ILC2s, as promoters of Th2 immunity, are of most interest in the context of asthma and allergic diseases. Recent studies have revealed the role ILC2s in IL-33 driven Th2 and Treg cell expansion in the lungs. Expression of the costimulatory molecule, OX40 ligand (OX4L), by ILC2 is critical for orchestrating an effective Th2 immune response and ILC2-derived IL-13 has been shown to potentiate memory Th2 cell responses by inducing dendritic cell release of Th2 attracting chemokine CCL17 (76, 77).

*Ex vivo* exposure of IL-33 expanded lung ILC2s to butyrate (0.5–1 mM) reveals a dose-dependent reduction *Il13* and *Il5* gene expression and IL-5 and IL-13 cytokine secretion (78). Notably, acetate and propionate treatment have no effect on ILC2 cytokine secretion. Butyrate suppression of IL33-induced IL-5 and IL-13 secretion has also been observed in both allergen-induced and naïve ILC2s (78). Butyrate also potently inhibits IL-5 and IL-13 secretion by IL-33-stimulated human ILC2s sorted from healthy donor PBMCs (78). Recall that butyrate only potently promoted apoptosis in IL-5-primed eosinophils (52), so this represents a distinct mechanism. In addition, butyrate does not alter IL-17A and IFN-γ production by *Rag2*^−/−^ ILC3s (78).

In *Alternaria alternata* models of allergic lung inflammation, both chronic systemic (6 weeks and 150 mM butyrate in drinking water) and local intranasal (50 μL of 10 mM butyrate daily) (78) administration of butyrate dampens disease severity. Attenuation of ILC2-mediated lung inflammation by butyrate is marked by decreased ILC2 secretion of IL-5 and IL-13 (78). Normally these cytokines act synergistically to induce lung eosinophilia, goblet cell hyperplasia, and airway hyperresponsiveness (79). Concurrent with a reduction in IL-5 and IL-13, total eosinophil counts in the BALF of butyrate-treated mice were significantly reduced (78). Prior work has also shown that butyrate induces Treg cell expansion. While Tregs can suppress ILC2-driven airway hyperreactivity and inflammation, it is intriguing that the attenuation of these features in the *A alternata* asthma model was not attributed to Treg expansion (80). Adoptive transfer of human PBMCs into NOD-SCID IL2rγ^−/−^ mice under conditions that promote human ILC2 (hILC2) development demonstrates that butyrate administered to the airways (i.n.) also attenuates the appearance of IL-5 and IL-13 producing hILC2s in response to intranasal IL-33 (78).

At the molecular level, butyrate epigenetically regulates ILC2 *Gata3* expression and limits ILC2 proliferation, but not survival (78). Again, these effects are independent of GPR41/43 (78). Mechanistically, it is likely that butyrate inhibits ILC2 function through histone deacetylase inhibition as both, the effects of dampened IL-5/-13 secretion and suppressed proliferation on IL-33-activated ILC2s, were recapitulated by exposure of ILC2s to the HDACi, trichostatin A (78). *In vitro*, butyrate and TSA significantly induce acetylation of histone H3 at concentrations at or above 0.5 mM and 1 nM, respectively (78). *Gata3* gene expression is downregulated in both mouse and human ILC2s treated with butyrate (78, 81). In addition, butyrate has been shown to modulate the overall metabolic activity of pulmonary ILC2s by decreasing the inherent ability of ILC2s to utilize both oxidative phosphorylation and glycolysis (81). Although epigenetic regulation is likely, the precise molecular mechanism underlying the inhibition of ILC2 proliferation and *Gata3* expression remains to be conclusively resolved. The chromatin landscape surrounding genes associated with ILC2 proliferation has yet to be explored and is a clear prerequisite for resolving the mechanism behind butyrate-induced amelioration of ILC2-driven AHR and inflammation.

### B Cells

The role of B cells in allergic lung inflammation and asthma has been explored extensively and it is well-known that Th2 driven disease has a profound effect on B cell maturation and effector functions (82). Specifically, IL-4 elaborated by innate cells and T cells potently stimulates B cell maturation and directed isotype switching to IgG1 and IgE. The Fc domains of these antibodies play key and profound roles in appropriate Th2 effector functions from myeloid cells including mast cells, basophils, and monocyte/macrophages. In addition, as class II MHC expressing cells, B cells are also well-positioned to function as antigen presenting cells during the early phase of allergic priming and their effects on initiating and amplifying Th2 cell responses have been demonstrated through *ex vivo* experiments and *in vivo* models of asthma (83). Conversely, the depletion of B cells with alpha-CD20 antibodies prior to HDM challenge results in reduced allergic inflammation with a characteristic decrease in CD4+CD44+ T cells, effector memory Th cells, eosinophils, and neutrophils. Together, this evidence suggests that B cells and humoral immunity play a critical role in the initiation and perpetuation of allergic asthma (83).

Recent findings have revealed new insights into the epigenetic effects of butyrate on B cell function. Mice fed a high fiber diet show a dose-dependent decrease in local and systemic antibody response with reductions in B cell activation-induced cytidine deaminase (AID/*Aicda*) and B lymphocyte-induced maturation protein 1 (Blimp-1/*Prdm1*) expression, and a decrease in both class-switched B cells and circulating antibodies specifically IgG1, IgA, and IgE (84). AID plays a critical functional role in both isotype switching, a critical step in the production of IgG1 and IgE, and somatic hypermutation that leads to antibody affinity maturation. Blimp-1, in contrast, orchestrates the maturation of activated B cells into terminally differentiated plasma cells and the corresponding switch from membrane bound to secretory immunoglobulin production. Thus, their inhibition effectively squelches the critical maturation of B cells required for an effective humoral immune response. It is intriguing that at low concentrations butyrate seems to enhance class-switch DNA recombination and increase AID and Blimp-1 expression *in vivo* (84). This could be due to concentration specific effects of butyrate on HDAC isoforms within B cells themselves or, alternatively, an effect of butyrate on other target cells required to produce cytokines that drive isotype switching and plasma cell maturation like IL-4 and IL-6, respectively.

In summary, the epigenetic mechanisms underlying butyrate-mediated modulation of intrinsic B cell function include the inhibition/silencing of genes involved in somatic hypermutation, class-switching, plasma cell differentiation, and development (84). Butyrate negatively regulates expression of *Aicda/*AID and *Prdm1/*Blimp-1 in mouse and human B cells resulting in a dose-dependent reduction in plasma cell differentiation and restraining class-switching to IgG, IgA, and IgE. Remarkably, the epigenetic-induced gene silencing effects of butyrate are achieved through selective upregulation of miRNAs that target *Aicda* and *Prdm1* 3'UTR (84).

## Conclusions

**Figure 4:**
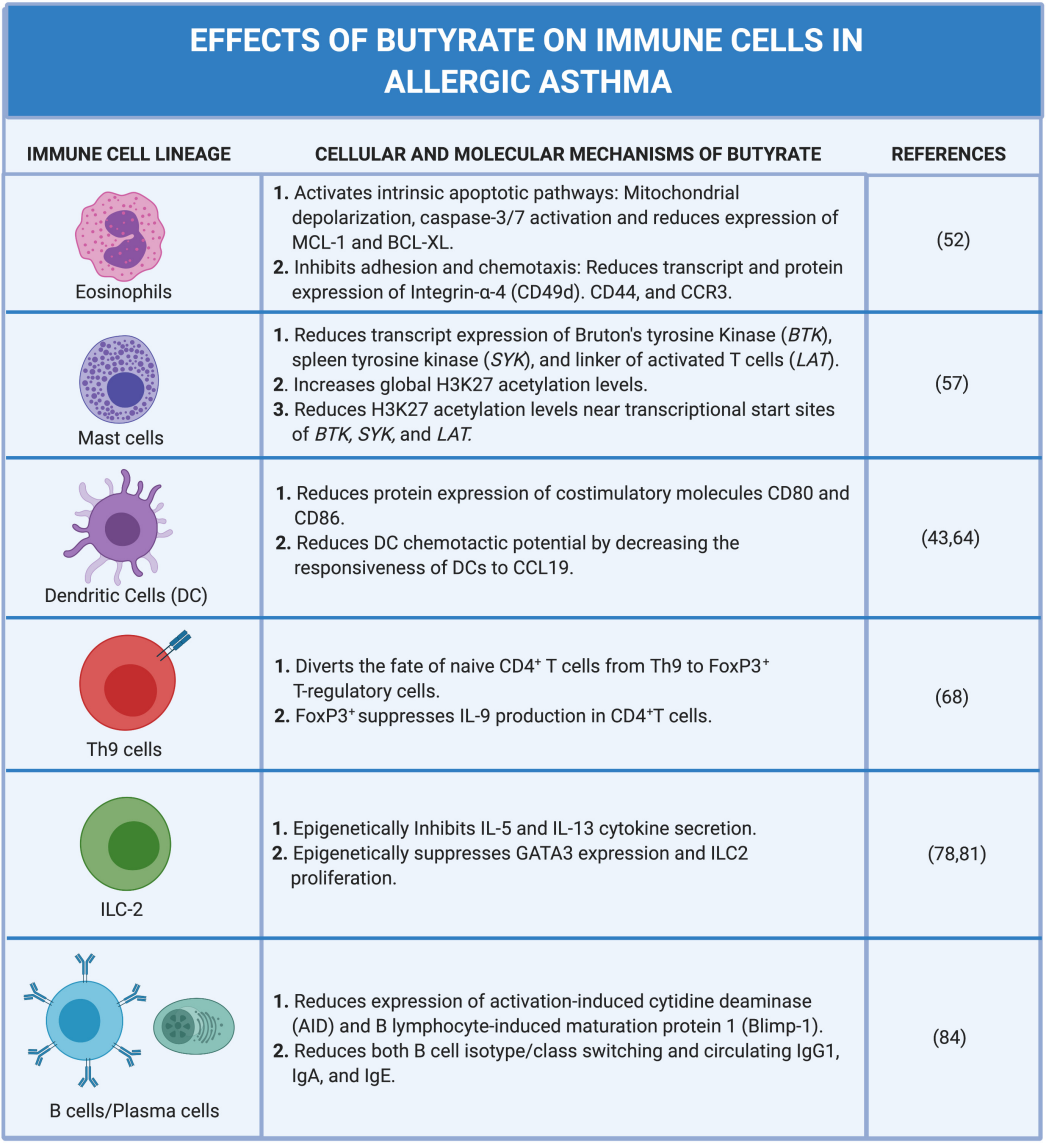
Cellular and molecular mechanisms of butyrate on immune cells in allergic asthma. Created with https://biorender.com/.

Allergic asthma is a complex inflammatory disease imitated by allergens and tissue damage and propagated by the coordinated activation and recruitment of several immune cell subsets across the innate and adaptive immune spectrum. While the full understanding of how SCFAs, in particular, butyrate, influence allergic airway disease pathology remains obscure, a recurring theme has emerged: Butyrate regulates immune cell behavior predominantly through epigenetic modification of cell fate and function (Figure 4). Such epigenetic control mechanisms may have a long-term impact on immune cell fate during embryonic development and therefore offers an attractive explanation for the observed narrow “window of opportunity” in early life where commensal bacteria and their metabolites can impact life-long allergic susceptibility and severity. In fact, this window of opportunity appears to end after the nursing stage in humans and mice. Most intriguing, peak influence may extend earlier, into the pre-natal development stage and depend on maternal commensals and their metabolites. Defining these mechanisms more precisely and establishing a causal link to butyrate (and/or other metabolites) will enable a clearer understanding of butyrate's influence on immune cell ontogeny. With evidence that butyrate impacts the epigenetic regulation of mature immune cell subsets it is natural to ask what role butyrate may have on shaping cell fate at much earlier stages in hematopoiesis. Despite the presumably low concentration of butyrate most hematopoietic stem and progenitor cells (HSPCs) may “see” in the bone marrow niche (and perhaps brief forays into circulation), butyrate has the potential to influence cell fates throughout the immune system.

Finally, while it is clear that butyrate can potently influence immune cell functions at mM concentrations present in the intestinal lumen, it remains difficult to explain the more widespread *in vivo* effects of butyrate on inflammatory disease in the lung and other peripheral tissues where the concentration of butyrate is 500-1,000-fold less. Since PBMCs circulate through the intestinal and hepatic supply, it may be that this short term but repeated exposure to high μM concentrations were sufficient to influence cell functions. Alternatively, sustained exposure to low μM levels of butyrate may provide enough HDAC inhibition to alter cell fate in peripheral tissues.

While dietary supplementation of butyrate attenuates lung inflammation in dysbiotic mouse models there are serious limitations to the potential therapeutic utility of butyrate in human asthma. First, and foremost, butyrate supplementation may be most effective perinatally, long before symptoms of allergic lung inflammation or atopic disease are detected. It is likely to be more practical to assess gut microbiota perinatally and identify safe and effective methods to optimize microbial communities for future health. Alternatively, more potent HDAC inhibitors, especially those with HDAC isoform-specificity could have therapeutic potential in established allergic lung disease. Regardless, it is essential to more fully understand how microbial-derived butyrate (and other metabolites) work to tune immune responses.

## Author Contributions

WY and MRH wrote the manuscript and designed the figures. YL, AC, MH, WM, and KM edited and assisted with the development of the manuscript throughout the writing process. All authors contributed to the article and approved the submitted version.

## Conflict of Interest

The authors declare that the research was conducted in the absence of any commercial or financial relationships that could be construed as a potential conflict of interest.
